# Monasnicotinates A–D, Four New Pyridine Alkaloids from the Fungal Strain *Monascus pilosus* BCRC 38093

**DOI:** 10.3390/molecules16064719

**Published:** 2011-06-07

**Authors:** Ming-Der Wu, Ming-Jen Cheng, Yi-Jen Yech, Yen-Lin Chen, Kai-Ping Chen, Ih-Sheng Chen, Ping-Hsun Yang, Gwo-Fang Yuan

**Affiliations:** 1 Bioresource Collection and Research Center (BCRC), Food Industry Research and Development Institute (FIRDI), Hsinchu 300, Taiwan; 2 School of Pharmacy, College of Pharmacy, Kaohsiung Medical University (KMU), Kaohsiung 807, Taiwan

**Keywords:** *Monascus pilosus* BCRC 38093, pyridine derivatives, NO production

## Abstract

Four new pyridine derivatives, monasnicotinates A–D (**1**–**4**) were isolated from the red yeast rice of *Monascus pilosus* BCRC 38093. Their structures were elucidated on the basis of physicochemical evidence, in-depth NMR spectroscopic analysis, and high-resolution mass spectrometry. Their inhibitory effects on NO production was also evaluated.

## 1. Introduction

*Monascus*-fermented rice (ang-kak, red koji) has been used for centuries as a natural food colorant and traditional medicine in oriental countries. The species of *Monascus* produced secondary metabolites such as pigments [1], monacolin K [2], γ-aminobutyric acid [3], dimerumic acid [4], and citrinin [5]. Several secondary metabolites from *Monascus* sp. have been found to have some beneficial pharmacological effects in decreasing blood pressure [6], lowering plasma cholesterol levels [2,7,8] and antibacterial activity [9]. In Taiwan, *M. purpureus*, *M. pilosus*, and *M. ruber*, are the common species used to make *Monascus*-fermented red rice, which contains some red pigments and physiological biological active metabolites. *M. pilosus* is one of the fungi traditionally used in food items in south China, Taiwan, Japan, Korea, Indonesia and other eastern countries. Many metabolites were identified from the *Monascus* species in previous studies [1,5,10,11,12,13,14,15,16,17,18,19], but knowledge of their biological or toxicological effects is limited. Therefore, characterization of the secondary metabolites of *Monascus* and their functionality still remain unclear and are worthy of examination. 

In a series of studies on the nitric oxide (NO) production inhibitory activity from natural sources, we were especially interested to realize the chemical constituents of red yeast rice, and *M. pilosus* BCRC 38093 has been found to be one of the active species. Chromatographic purification of the AcOEt-soluble fraction of a 95% EtOH extract of the red yeast rice produced by *M. pilosus* BCRC 38093 led to the isolation of four new pyridine derivatives, monasnicotinates A–D (**1**–**4**). The structural elucidation of the new natural compounds and the inhibitory effects on NO production by macrophages of the isolates are described herein.

**Figure 1:**
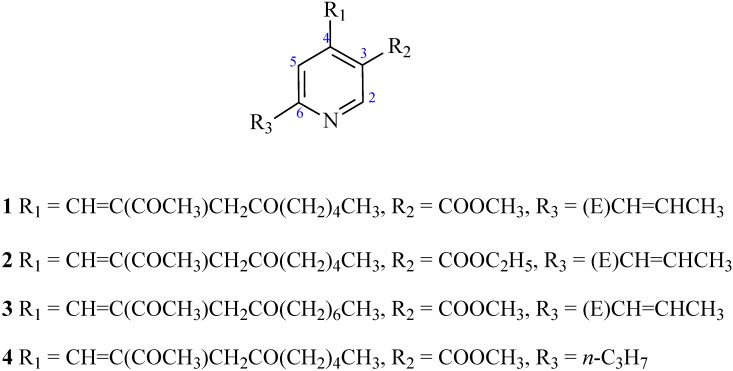
Chemical structures of compounds **1**–**4**.

## 2. Results and Discussion

Compound **1**, obtained as a yellowish oil, had the molecular formula C_21_H_27_NO_4_, requiring nine degrees of unsaturation, as determined by HR-ESI-MS data [*m/z* 380.1837 ([M+Na]^+^; calc. 380.1835)] in combination with its ^1^H-NMR, ^13^C-NMR and DEPT data. The IR spectrum revealed the presence of multiple carbonyls (1712, 1668 cm^-1^), one of which was by UV spectrum analysis (λ_max_ 253, 280 and 330 nm) in conjugation with a pyridine. 

The ^1^H-/^13^C-NMR spectra (Table 1) indicated seven quaternary C-atoms, five CH, five CH_2_, and four Me groups. In the ^1^H-NMR spectrum, there were typical signals for one OMe groups at δ_H_ 3.92 (s, MeO-C(12)), one acetyl moiety at δ 2.51 (3H, s, Me-15), signals of α-methylene protons of a ketone at δ 2.54 (2H, d, *J* = 7.4 Hz, CH_2_-18) and 3.27 (2H, s, CH_2_-16), one β-methylene resonance of a ketone at δ 1.59 (2H, m, CH_2_-19), two signal for a pyridine olefinic proton at δ 7.33 (1H, s, H-5) and 9.13 (1H, s, H-2), as well as one (*E*)-double bond signals at δ 6.54 (1H, br d, *J* = 16.0 Hz, H-7) and 6.93 (1H, dq, *J* = 16.0, 7.0 Hz, H-8), indicating that **1** was probably a pyridine ring moiety possessing a conjugated carbonyl ester group. The carbons of the pyridine derivative were assigned, from ^13^C-NMR and DEPT experiments (Table 1), there were resonances for three C=O functions [δ 198.8 (α,β-unsaturated C=O group); 165.5 (ester C=O group), and 208.7 (C=O)], one C=C bond [δ_C_ 130.2, 135.4], one vinyl methyl carbon [δ_C_ 18.6], one methoxyl groups [δ_C_ 52.3], one acetyl methyl moiety [δ_C_ 25.4], and three aliphatic methylenes C-atoms [δ_C_ 20.0, 31.0, 34.6]. 

**Table 1:** ^1^H- (400 MHz) and ^13^C-NMR (100 MHz) data of **1** and **2** in CDCl_3_. δ in ppm, J in Hz.

No.	**1**			**2**	
	δ_H_	δ_C_		δ_H_	δ_C_
2	9.13 (*s*)	151.8		9.14 (*s*)	151.7
3		121.1			121.4
4		145.7			145.7
5	7.33 (*s*)	120.1		7.24 (*s*)	120.1
6		159.4			159.2
7	6.54 (*dd*, *J* = 16.0, 1.8)	130.2		6.54 (*dd*, *J* = 16.0, 2.0)	130.1
8	6.93 (*dq*, *J* = 16.0, 7.0)	135.4		6.92 (*dq*, *J* = 16.0, 7.0)	135.5
9	1.96 (*dd*, *J* = 7.0, 1.8)	18.6		1.95 (*dd*, *J* = 7.0, 2.0)	18.7
10		165.5			165.1
MeO-12	3.92 (*s*)	52.3			
12				4.36 (*q*, *J* = 7.2)	61.4
13	8.13 (*s*)	140.9			
Me-13				1.39 (*t*, *J* = 7.2)	14.2
C-14		136.9			141.0
C-15		198.8			136.9
Me-15	2.51 (*s*)	25.4			
Me-16				2.50 (*s*)	25.4
16	3.27 (*s*)	40.6			198.8
17		208.7		3.27 (*s*)	40.7
18	2.54 (*d*, *J* = 7.4)	43.2			208.7
19	1.59 (*m*)	23.4		2.54 (*t*, *J* = 6.8)	43.2
20	1.32 (*m*)	31.3		1.59 (*m*)	23.5
21	1.27 (*m*)	22.4		1.32 (*m*)	31.3
Me-22	0.88 (*t*, *J* = 6.8)	13.9			
22				1.32 (*m*)	22.4
Me-23		–		0.87 (*t*, *J* = 7.2)	13.9

The above observation accompanied by the ^1^H,^1^H-COSY, and NOESY (Figure 1) spectrum of **1** was constructed by the aid of HMBC spectrum (Figure 2). Thus, the structure of **1** was determined to be methyl 4-((*E*)-2-acetyl-4-oxonon-1-enyl)-6-((*E*)-prop-1-enyl)nicotinate, and designated monasnicotinate A. 

**Figure 1:**
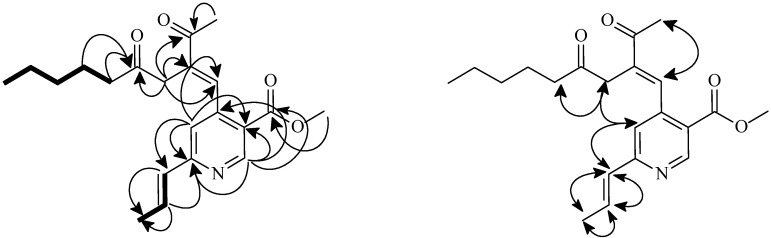
Key COSY (▬), NOESY (H↔H), and HMBC (H→C) correlations of **1**.

**Figure 2:**
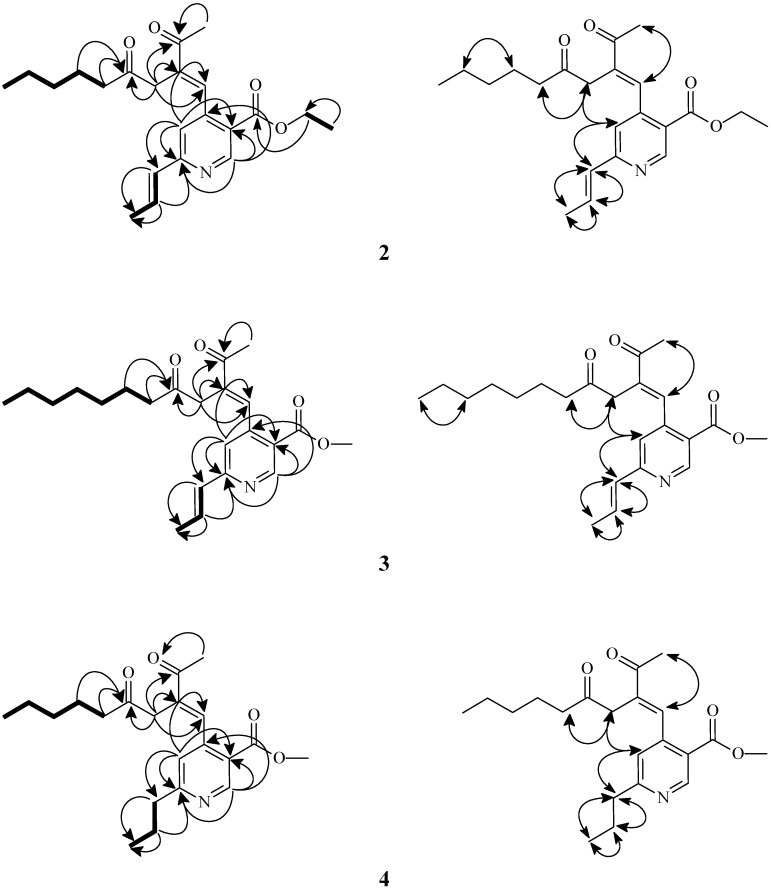
Key COSY (▬), NOESY (H↔H), and HMBC (H→C) correlations of **2–4**.

The ^1^H/^13^C-NMR spectra of compounds **2–4** (Table 1 and Table 2) were similar to those of the above compound **1**, except that the substitution patterns on the pyridine moiety were different (Figure 1). The structures were further confirmed by ^13^C-NMR, DEPT, COSY, NOESY (Figure 2), HSQC, and HMBC (Figure 2) experiments. Thus, the structures of **2–4** were elucidated to be ethyl 4-[(*E*)-2-acetyl-4-oxonon-1-enyl]-6-[(*E*)-prop-1-enyl]nicotinate, methyl 4-[(*E*)-2-acetyl-4-oxoundec-1-enyl]-6-[(*E*)-prop-1-enyl)]- nicotinate and (*E*)-methyl 4-(2-acetyl-4-oxonon-1-enyl)-6-propylnicotinate and named monasnicotinates B**–**D, respectively. 

**Table 2:** ^1^H- (400 MHz) and ^13^C-NMR (100 MHz) data of **3** and **4** in CDCl_3_. δ in ppm, J in Hz.

	**3**			**4**	
	δ_H_	δ_C_		δ_H_	δ_C_
2	9.14 (*s*)	151.8		9.16 (*s*)	150.9
3		121.1			121.5
4		145.8			146.2
5	7.24 (*s*)	120.2		7.23 (*s*)	122.8
6		159.4			166.4
7	6.54 (*dq*, *J* = 15.6, 2.0)	130.2		2.83 (*t*, *J* = 7.2)	39.8
8	6.92 (*dq*, *J* = 15.6, 7.0)	135.6		1.75 (*sext*, *J* = 7.2)	22.7
9	1.95 (*dd*, *J* = 7.0, 2.0)	18.7		0.94 (*t*, *J* = 7.2)	13.7
10		165.6			165.3
MeO-12	3.92 (*s*)	52.4		3.93 (*s*)	52.5
13	8.12 (*s*)	140.9		8.13 (*s*)	140.5
14	–	137.0			137.2
15	–	198.8		–	198.7
Me-15	2.51 (*s*)	25.4		2.51 (*s*)	25.4
16	3.27 (*s*)	40.7		3.26 (*s*)	40.7
17	–	208.7			208.4
18	2.53 (*d*, *J* = 7.2)	43.8		2.52 (*t*, *J* = 7.2)	43.2
19	1.58 (*m*)	23.8		1.57 (*m*)	23.4
20	1.26 (*m*)	31.6		1.26 (*m*)	31.3
21	1.26 (*m*)	22.6		1.26 (*m*)	22.4
22	1.26 (*m*)	29.1		0.88 (*t*, *J* = 7.2)	13.9
23	1.26 (*m*)	22.6		–	–
24	0.87 (*t*, *J* = 7.2)	14.1		–	–

We examined the inhibitory effects of these compounds **1**–**4** on the production of NO induced by LPS. Compound **1**, **3**, and **4** showed stronger inhibition on NO production (*IC_50_* = 6.95, 8.34, and 9.42 μg/mL) than quercetin, used as a positive control (*IC_50_* = 11.01 μg/mL). Cytotoxic effects of compounds **1**, **3,** and **4** were measured using the MTT assay and these compounds did not show any cytotoxic effects. 

## 3. Experimental

### 3.1. General

TLC: silica gel 60 F_254_ precoated plates (Merck). Column chromatography (CC): silica gel 60 (70-230 or 230-400 mesh, Merck). M.p.: Yanaco micro-melting point apparatus; uncorrected. UV Spectra: Jasco UV-240 spectrophotometer; λ_max_ (log ε) in nm. Optical rotation: Jasco DIP-370 polarimeter; in CHCl_3_. IR Spectra: Perkin-Elmer-2000 FT-IR spectrophotometer; ν in cm^-1^. ^1^H-, ^13^C- and 2D-NMR spectra: Varian-Gemini-200, Varian-Unity-Plus-400 and Varian-Mercury-400 spectrometers; δ in ppm rel. to Me_4_Si, J in Hz. GC-MS: Trace GC/POLARIS Q Thermo Finnigan; in m/z (rel. %). EI-MS: VG-Biotech Quatro-5022 mass spectrometer; in m/z (rel. %). ESI- and HR-ESI-MS: Bruker APEX-II mass spectrometer; in m/z.

### 3.2. Fungal Material

*Monascus pilosus* BCRC 38093 was used throughout this study, and specimens deposited at the Bioresource Collection and Research Center (BCRC) of the Food Industry Research and Development Institute (FIRDI). 

### 3.3. Fungal Material

BCRC 38093 was maintained on potato dextrose agar (PDA; Difco). The strain was cultured on PDA slants at 25°C for 7 days and then the spores were harvested by sterile water. The spores (5 × 10^5^) were seeded into 300 mL shake flasks containing 50 mL RGY medium (3% rice starch, 7% glycerol, 1.1% polypeptone, 3% soybean powder, 0.1% MgSO_4_, 0.2% NaNO_3_), and cultivated with shaking (150 rpm) at 25 °C for 3 days. After the mycelia enrichment step, an inoculum mixing 100 mL mycelia broth and 100 mL RGY medium was inoculated into plastic boxes (25 cm × 30 cm) containing 1 kg sterile rice and cultivated at 25 °C for producing red yeast rice. At day 7, 150 mL RGY medium was added for maintaining the growth of cells. After 28 days of cultivation, the red yeast rice was harvested and lyophilized for metabolites extraction. 

### 3.4. Extraction and ISOLATION

The dried red yeast rice extracted three times with 95% EtOH (6 L) at rt. The ethanol syrup extract was partitioned between EtOAc and H_2_O (1:1) (10 L) to afford the EtOAc soluble fraction (76 g). The EtOAc-soluble fraction was chromatographed over silica gel (75 g, 70-230 mesh) and eluted with hexane/EtOAc: 12:1, 10:1, 8:1, 6:1, 4:1, 2:1, 1:1, EA, EtOAc/MeOH: 8:1, 6:1, 4:1, 2:1, 1:1, and MeOH (each 1 L) to afford thirty-two fractions. Fr. 10 was purified by prep. TLC (hexane/EtOAc, 2:1) to give **1** (36.4 mg). Fr. 11 was purified by preparative TLC (hexane/EtOAc, 2:1) to give **2** (7.8 mg) and **3** (39.2 mg). Fr. 14 was further purified by silica gel column chromatography eluting with hexane/EtOAc: 8:1, 6:1, 4:1, 2:1, 1:1 and EtOAc (each 500 mL) to yield seven fractions (frs 14.1 to 14.7). **4** (3.5 mg) was furnished from fraction 14.3 by prep. TLC (hexane/EtOAc, 2:1).

### 3.5. Biological Assay

*Determination of Nitric Oxide Production*. The murine macrophage cells RAW264.7 (BCRC 60001 = ATCC TIB-71) were transferred to 96-well plates at a density of 1x10^5^ cell/well. After 24 hr incubation, the cells were stimulated with 1 μg/mL of LPS (Sigma, Cat no: L-2654) for 24 hr in the presence or absence of the compounds (0, 1, 5, 10 and 20 μg/mL) tested. As a parameter of NO synthesis, nitrite concentration was measured in the supernatant of RAW264.7 cells by the Griess reagent [1:1 mixture of 1% sulfanilamide and 0.1% *N*-(1-naphthyl)ethyl-enediamine dihydrochloride, each in 2.5% phosphoric acid solution] in a 96-well plate, and incubated for 10 min at room temperature. Nitrite concentration was determined by measuring the absorbance at 540 nm using an ELISA plate reader (*μ* Quant) [20]. All tests were run in triplicate and averaged. The data were expressed as a mean of three experiments. Statistical comparisons were carried out the Student’s *t*-test for paired values.

*Determination of cell viability*. The cell viability was assessed using a MTT [3-(4,5-dimethylthiazol-2-yl)-2,5-diphenyltetrazolium bromide, Merck KGaA, Damstadt, Germany]-based colorimetric assay, as previously described [21]. After sampling the supernatant for the NO assay, 100 μL of fresh medium containing 0.5 mg/mL of MTT was added to each well and incubated for 3 hr at 37 °C. The medium was then removed and the violet for mazan crystals in the viable cells were dissolved in dimethyl sulfoxide. The absorbance of each well was then read at a wavelength of 540 nm using microplate reader (*μ* Quant, Bio-TEK instruments INC). 

### 3.6. Spectral Data

*Monasnicotinate A* (**1**). Yellowish oil. UV λ_max_ (MeOH): 253, 280, 330. IR ν_max_ (Neat): 1668, 1712 (C=O). ^1^H- and ^13^C-NMR: see Table 1. ESI-MS: 380 ([*M*+Na]^+^). HR-ESI-MS: 380.1837 ([*M*+Na]^+^, C_21_H_27_NaNO_4_^+^; calcd. 380.18353). 

*Monasnicotinate B* (**2**). Yellowish oil. UV λ_max_ (MeOH): 248, 275, 338. IR ν_max_ (Neat): 1676, 1716 (C=O). ^1^H- and ^13^C-NMR: see Table 1. ESI-MS: 394 ([*M*+Na]^+^). HR-ESI-MS: 394.1994 ([*M*+Na]^+^, C_22_H_29_NaNO_4_^+^; calcd. 394.1998).

*Monasnicotinate C* (**3**). Yellowish oil. UV λ_max_ (MeOH): 251, 282, 327. IR ν_max_ (Neat): 1672, 1724 (C=O). ^1^H- and ^13^C-NMR: see Table 2. ESI-MS: 408 ([*M*+Na]^+^). HR-ESI-MS: 408.2151 ([*M*+Na]^+^, C_22_H_29_NaNO_4_^+^; calcd. 408.2153). 

*Monasnicotinate D* (**4**)**.** Yellowish oil. UV λ_max_ (MeOH): 245, 271, 328. IR ν_max_ (Neat): 1665, 1716 (C=O). ^1^H- and ^13^C-NMR: see Table 2. ESI-MS: 382 ([*M*+Na]^+^). HR-ESI-MS: 382.1994 ([*M*+Na]^+^, C_21_H_29_NaNO_4_^+^; calcd. 382.1994).

## 4. Conclusions

In this study, we focused on the minor secondary metabolites appearing in the EtOAc-soluble fraction of a 95% EtOH extract of the red yeast rice produced by *Monascus purpureus* BCRC 38093. Four new natural metabolites **1**–**4** were found in this study. In the *in vitro* NO production inhibitory assay, isolates **1**, **3**, and **4** showed stronger inhibition on NO production with IC_50_ value of 6.95, 8.34, and 9.42 μg/mL respectively, while showing no cytotoxicity to normal cells at the same concentration.
